# Numerical Simulation of Lost-Foam Casting for Key Components of A356 Aluminum Alloy in New Energy Vehicles

**DOI:** 10.3390/ma17102363

**Published:** 2024-05-15

**Authors:** Chi Sun, Zhanyi Cao, Yanzhu Jin, Hongyu Cui, Chenggang Wang, Feng Qiu, Shili Shu

**Affiliations:** 1 Key Laboratory of Automobile Materials of Ministry of Education, Department of Materials Science and Engineering, Nanling Campus, Jilin University, Changchun 130025, China; sunchi17@mails.jlu.edu.cn (C.S.); caozy@jlu.edu.cn (Z.C.); cuihy1621@jlu.edu.cn (H.C.); 2 FAW Foundry Co., Ltd., Changchun 130013, China; jinyanzhu2009@163.com (Y.J.); wcg_faw@sina.com (C.W.); 3 School of Mechanical and Aerospace Engineering, Nanling Campus, Jilin University, Changchun 130025, China

**Keywords:** numerical simulation, lost-foam casting, casting porosity

## Abstract

The intricate geometry and thin walls of the motor housing in new energy vehicles render it susceptible to casting defects during conventional casting processes. However, the lost-foam casting process holds a unique advantage in eliminating casting defects and ensuring the strength and air-tightness of thin-walled castings. In this paper, the lost-foam casting process of thin-walled A356 alloy motor housing was simulated using ProCAST software (2016.0). The results indicate that the filling process is stable and exhibits characteristics of diffusive filling. Solidification occurs gradually from thin to thick. Defect positions are accurately predicted. Through analysis of the defect volume range, the optimal process parameter combination is determined to be a pouring temperature of 700 °C, an interfacial heat transfer coefficient of 50, and a sand thermal conductivity coefficient of 0.5. Microscopic analysis of the motor housing fabricated using the process optimized through numerical simulations reveals the absence of defects such as shrinkage at critical locations.

## 1. Introduction

The motor housing, as one of the most critical components in new energy vehicles, requires excellent mechanical performance and intricate thin-wall structures to meet the needs of protecting the motor and ensuring efficient heat dissipation for the motor [1]. Aluminum alloys have received widespread attention in the aerospace and vehicle weight reduction fields due to their low density and excellent corrosion resistance properties [2]. The aluminum alloy motor housing serves as a core casting in the powertrain of new energy vehicles [3,4]. Its top and bottom parts are respectively connected to the inverter and the reducer, and it connects to the vehicle’s main shaft bearings through bearing liners. The primary technical requirement of the motor casing is to secure and protect the internal motor, shielding it from external forces or environmental contaminants. Additionally, the motor housing also serves to dissipate the heat generated by the motor. The sidewalls of the motor housing typically feature a circumferential cooling water jacket. The complex thin-wall structure poses a challenge in casting. Due to the pressure exerted on the water jacket during operation, even minor casting defects could lead to subsequent coolant leakage. Therefore, achieving the highest yield requires continuous refinement of the casting process and adjustment of casting parameters to optimize the process.

Lost-foam casting is a highly precise and versatile casting process used for producing various metal parts and artwork [5,6,7]. Its advantages include the ability to create complex shapes, high precision, and excellent surface quality castings. Lost-foam casting does not require complex process designs like gravity casting nor extensive pouring equipment and mold expenses like low-pressure casting. It offers a high cost-effectiveness and operability in actual production. These qualities make lost-foam casting the preferred method for producing high-quality metal products in many industries [8]. Given that lightweighting is a pivotal approach for energy conservation and emission reduction in hybrid vehicles, as well as for extending the driving range of electric vehicles, the aluminum alloy stands out for its outstanding mechanical properties and corrosion resistance [9,10]. This enables the A356 alloy to fulfill performance requirements while simultaneously reducing vehicle weight to enhance driving range [11,12]. Therefore, the main research direction for manufacturing motor casings is the adoption of aluminum alloy lost-foam casting.

In the handling of lost-foam castings characterized by intricate geometries and slender wall thicknesses, particularly exemplified by typical power system components such as motor housings, the predominant challenge resides in avoiding defects at the thin-walled and complex-shaped locations during solidification. A356 is a hypoeutectic aluminum–silicon alloy, and its solidification behavior is influenced by various factors, including alloy composition, solidification temperature, solidification rate, and any added alloying elements [13,14]. Generally, A356 aluminum alloy has a wide solidification range. During solidification, grains form and gradually grow from the liquid phase [15]. The grain growth rate is influenced by solidification temperature and alloy chemical composition [16]. As the temperature decreases, certain regions within the alloy begin to solidify. These initially solidified regions form primary phases. As the temperature further decreases, eutectic reactions occur in the alloy, where the liquid simultaneously transforms into two solid phases. In A356 aluminum alloy, the eutectic phase is typically composed of a eutectic mixture of aluminum and silicon, known as α-Al and β-Si phases. During solidification, the volume of the melt continually contracts, which can lead to shrinkage porosity defects due to inadequate feeding. The eradication of defects arising during the manufacturing process remains an inherently intricate undertaking for components showcasing such pronounced features. Consequently, within the realm of lost-foam casting, the imperative lies in the precise simulation of the solidification process, with a specific emphasis on accurately forecasting latent defect locations. As a crucial analytical method, numerical simulation comprises various steps, including geometric modeling, flow simulation, heat transfer analysis, solidification analysis, and optimization design. Through multiple iterations of numerical simulation for process optimization, it not only visualizes the otherwise invisible behaviors of melt during casting but also maximizes the presentation of intricate details of the melt [17,18].

The shape of the melt front in lost-foam casting is very different from it in other casting methods [19,20]. Researchers improved the accuracy of numerical simulations of aluminum alloy lost-foam casting by refining the built-in models in Flow3D, making the simulation results closer to experimental data [18]. Defects produced during cooling in lost-foam casting are strongly related to the volume of the isolated liquid phase region [10,21]. Regrettably, limited research has been conducted to simulate diverse solidification conditions to optimize the process.

This study adjusted the solidification parameters of the A356 alloy according to its solidification characteristics. Comprehensive numerical simulations of motor housing preparation using aluminum alloy lost-foam casting were conducted using ProCAST software (2016.0), encompassing a total of nine experimental groups. Through a thorough analysis encompassing filling sequences, solidification kinetics, displacement fields, and prospective defect localizations, a systematic exploration of pivotal factors within the manufacturing process was undertaken. By means of scrutinizing the defect volume content within the range of these nine simulation sets, an optimal amalgamation of process parameters was successfully ascertained. Furthermore, through practical process validation, steadfast technical support has been provided for the forthcoming practical implementation of lost-foam casting production, facilitating the design and optimization of manufacturing processes.

## 2. Preprocess

### 2.1. Mathematical Models for Lost-Foam Casting Simulation

The filling process of the lost-foam casting technique mainly consists of two stages: the vaporization of the foam and the advancement of the melt. After the melt enters the mold cavity, the foam undergoes heating-induced decomposition and vaporization, creating a back pressure that maintains a certain distance between the melt and the undecomposed foam. However, as the gas inside the cavity is squeezed out, the melt continues to advance and occupies the space previously occupied by the vaporized foam. In the simulation of the filling process, the following assumptions are commonly made:

(1) Both gas and melt are treated as incompressible fluids.
(2) After solid foam undergoes thermal decomposition, it does not affect the heat transfer process.
(3) The foam undergoes direct decomposition from a solid to a gaseous state without any intermediate states.
(4) The flow of melt is assumed to be laminar since the overall filling velocities are slow, resulting in a low Reynolds number.

Description of the filling process for lost-foam casting in ProCAST can be achieved through certain parameters. As for the model of foam decomposition, the FOAMHTC (cal/cm^2^·°C·s) is used to describe the heat transfer coefficient at a distance of 1 cm from the melt front. The BURNZONE (cm) is used to describe the maximum distance traveled by the foam undergoing thermal decomposition from the melt front within a unit of time. The interface heat transfer between the foam and the melt is equal to the value of FOAMHTC/BURNZONE. The gas interface is unstable during decomposition, potentially leading to situations where the foam may come into contact with the melt. Therefore, it is necessary to set a maximum value for FOAMHTC to ensure that the calculation is feasible. The volume of foam decomposed per unit time can be calculated based on the heated foam volume and the total heat absorbed by this portion of foam. Regarding the model for the advancement of the melt, the magnitude of the back pressure is obtained by calculating the pressure of the gas within the cavity. The gases generated during the decomposition of expanded polystyrene include those originally trapped within the structure and gases produced from the complete breakdown of polystyrene.

Like many metals, the density of aluminum alloy melt increases as it cools. This manifests macroscopically as volume shrinkage during solidification, where the space vacated by the shrinkage is filled by the surrounding un-solidified melt. Meanwhile, solidification always progresses gradually from the outside inward, so the final solidified portions will face situations where volume needs to shrink, but there is no melt to replenish it. Temperature field simulation can calculate the temperature distribution of the melt under different cooling conditions, thus determining the location of the final solidified portion, known as the isolated liquid phase region. Shrinkage usually occurs in this region.

### 2.2. Three-Dimensional Model

**Figure 1:**
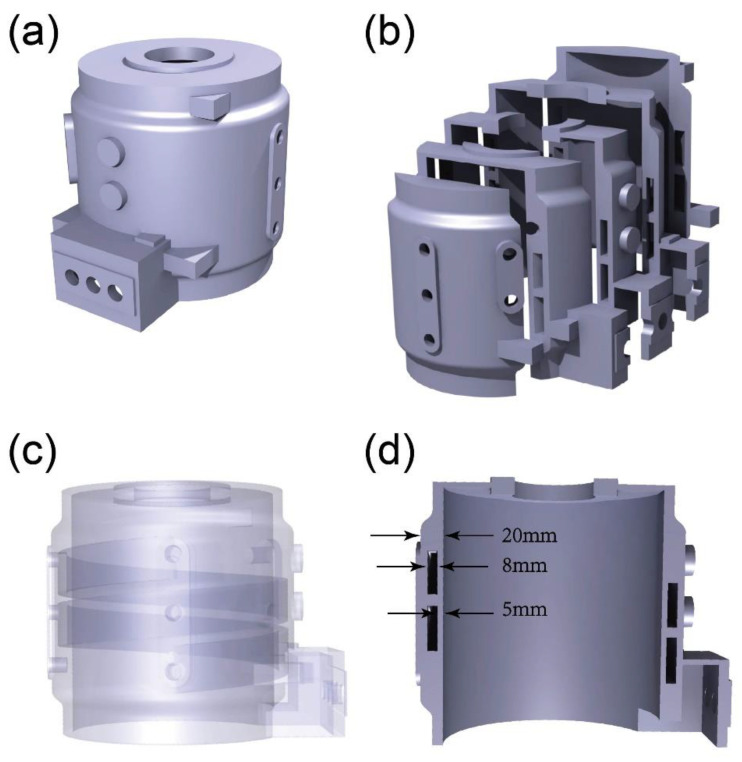
Motor housing 3D model in (**a**) overview perspective, (**b**) sectional perspective, (**c**) perspective view, and (**d**) cross-sectional view.

**Figure 2:**
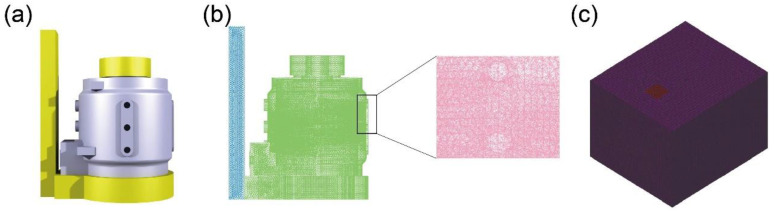
(**a**) Pouring system and mesh division of (**b**) casting and (**c**) mold.

As shown in Figure 1a, the three-dimensional model of the motor housing was constructed using CATIA. The dimensions of the motor housing are 303 × 260 × 260 mm, with a mass of 8.3 kg. The entire casting is barrel-shaped, with a water-cooling jacket embedded inside the side walls, as shown in Figure 1b. Figure 1c shows that the channels for water circulation hollow out the thickest part of the casting, originally intended to be the side wall. After hollowing out, the wall thickness is only 5 mm, making it the thinnest part of the casting. Figure 2a displays the casting system. The gray part represents the casting, while the yellow part represents the gating system and risers. This casting system is designed based on the structural characteristics of the motor casing. Compared to the central position where the water jacket is located, the upper and lower parts of the motor casing belong to thick and massive areas, resulting in slower solidification rates. Therefore, it is necessary to ensure that there is sufficient feeding and shrinkage compensation during the solidification of the upper and lower parts. Hence, larger risers with thicker shapes are added to these two sections. As shown in Figure 2b,c, the entire model, including the sandbox, was divided into finite element meshes, with a total of 3,655,882 mesh elements. Finer meshes are applied in critical regions of the casting to ensure the authenticity and reliability of the model. Additionally, finer element meshes can better capture the boundary shapes during the filling of the melt, which is beneficial for process improvement.

### 2.3. Process and Simulation Parameters

Considering the structural characteristics of the motor casing, risers with volumes of 628 and 1723 cm^3^ are respectively placed at the top and bottom of the motor housing. Additionally, the inlet of the gating system is positioned at the thickest section of the lower riser to ensure sufficient pressure of the melt towards critical locations of the casting during solidification, facilitating adequate feeding and shrinkage compensation. The pouring temperature range is set between 700 °C and 740 °C. The pouring speed is approximately 700 cm^3^ per second. This study utilized A356 aluminum alloy and expanded polystyrene with a density of 20 kg/m^3^ to fabricate the motor housing and its pattern. The primary parameters of A356 alloy in Table 1 for each material were obtained from the material database in the ProCAST software. The aggregate for the expendable mold casting coating consisted of 200-mesh bauxite clinker and graphite particles. By adjusting the proportions, the thermal conductivity of the coating could be significantly altered. Based on empirical evidence, when using alumina clinker exclusively, setting the interface heat transfer coefficient between the melt and sand mold to 50 yields highly accurate simulation results. Conversely, when employing graphite solely as the refractory aggregate, the interface heat transfer coefficient should be set to 200. When utilizing equal volumes of both materials, the interface heat transfer coefficient should be set to 125. The sand for mold contains silica sand and iron-made sand with a similar granularity. Similarly, we can alter the thermal conductivity of the mold sand by adjusting the proportions of the two types of sand. When the ratio of silica sand to iron sand is 1:1, the thermal conductivity coefficient of the sand is 1.25. When the ratio is adjusted to 7:3 or 3:7, the thermal conductivity coefficients of the sand are 0.5 and 2, respectively. As shown in Table 2, the experiment employed an orthogonal experimental design method with three factors and three levels.

## 3. Simulation Results and Discussion

### 3.1. Flow Field in Filling Process

**Figure 3:**
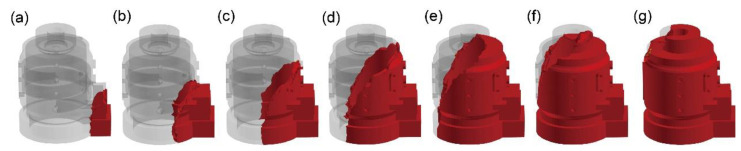
Filling process of lost-foam casting of Group 1 at 0.7 s (**a**), 1.8 s (**b**), 2.9 s (**c**), 4.0 s (**d**), 5.1 s (**e**), 6.2 s (**f**), and 7.3 s (**g**).

Figure 3 illustrates the filling process of the melt in Group 1. The pouring temperature is 700 °C, the thermal conductivity of the sand mold is 0.5 W/m·K, and the heat transfer coefficient of the coating is 50 W/m·K. As shown in Figure 3a, the melt enters the cavity filled with EPS foam from the sprue and gradually occupies the cavities formed after the decomposition of EPS. As illustrated in Figure 3b, unlike gravity casting, due to the presence of gas backpressure, the component of melt filling velocity in the gravity direction is not significantly greater than the component in the direction perpendicular to the gravity on the filling initiation stage. As depicted in Figure 3c, since the motor housing has a symmetrical structure and the sprue is located on the symmetrical surface, the overall filling symmetry of the melt during casting is observed in a left–right symmetric manner. Illustrated in Figure 3d, the melt interface in the lost-foam casting tends to be smooth, and turbulent flow is generally absent in the casting of thin-walled components. As shown in Figure 3e,f, the endpoint of melt filling is almost at the farthest point from the sprue.

**Figure 4:**
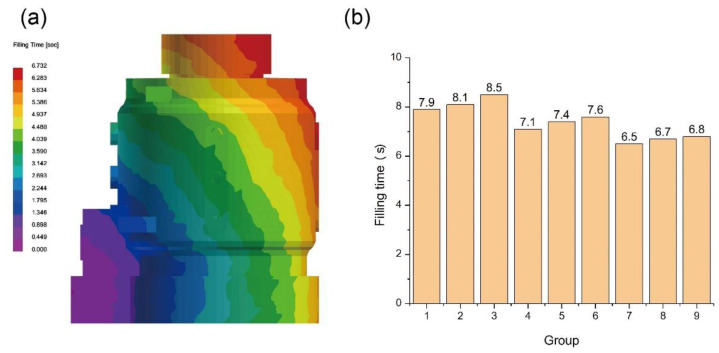
(**a**) Position of the liquid metal front at different times during the filling process and (**b**) filling time of different Groups.

The filling process of lost-foam casting is often associated with parameters such as pouring temperature, melt pressure, EPS density, casting geometry, and gating system location. As the filling time generally lasts only a few seconds, with the melt flowing continuously, the cooling parameters of the casting typically exert minimal influence on the filling process. In the scenario where both the pouring system and materials remain unchanged, the heat transfer coefficient of coatings and the thermal conductivity of sand have minimal impact on the filling process. Consequently, only the pouring temperature affects the filling time to some extent without altering the filling sequence. Figure 4a shows the statistically obtained filling volume per second, generally exhibiting the characteristic of a slow start and finish, with a faster speed in the middle. This is related to the expulsion speed of gas within the cavity. During the casting process, the melt exposes the coating near its leading edge in the cavity, serving as a means of venting. This indicates that the larger the perimeter ratio of the liquid metal front, the faster the gas expulsion rate, resulting in lower backpressure on the melt and, consequently, faster casting speed. Figure 4b illustrates the filling time of melt under nine different parameter sets. The filling speed increases with the rise in pouring temperature, with the average filling speed of Groups 7, 8, and 9 being 1.2 s faster than that of Groups 1, 2, and 3. This is attributed to the increased heating of the EPS as the pouring temperature rises. Not only does this accelerate the decomposition rate of EPS, but it also enhances the completeness of the decomposition reaction, leading to a higher rate of discharge of decomposition products. When the pouring temperature remains the same, the filling time is slightly influenced by the heat transfer coefficient of the coating. As the heat transfer coefficient of the coating increases, the filling time also slightly increases. This is due to the intimate relationship between the decomposition of EPS and the temperature at the liquid metal front. The higher the temperature at the liquid metal front, the more heat the EPS receives per unit time, resulting in higher decomposition rates and completeness. Due to the cooling state of the melt in lost-foam casting before reaching the front position, the heat transfer characteristics of the coating typically affect the temperature at the liquid metal front. This results in a slight reduction in the filling speed of the melt during the later stages of filling when using coatings with better thermal conductivity.

In summary, the casting process of the LFC in this study exhibits characteristics of diffusion-type filling. The filling speed is significantly influenced by the perimeter ratio of the melt front. Unlike gravity casting, the filling process remains stable even though the inlet is not at the lowest point, and it is hardly affected by the direction of gravity. Upon completion of the filling, the temperature distribution is uniform.

### 3.2. Temperature Field in Solidification Process

**Figure 5:**
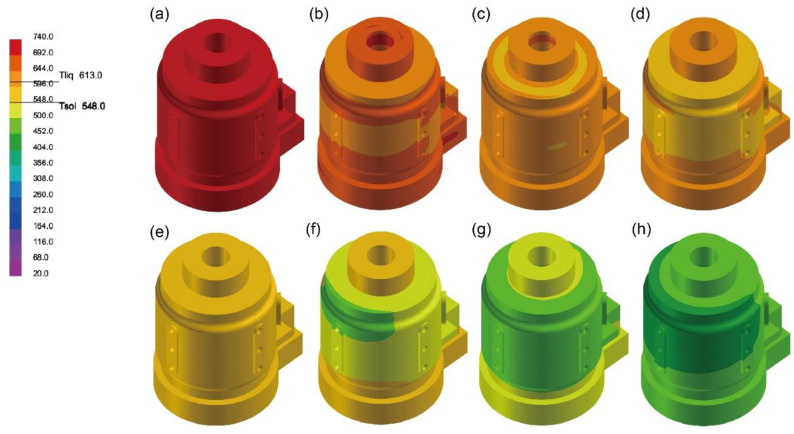
Solidification process of lost-foam casting of Group 1 at 0 s (**a**), 105 s (**b**), 180 s (**c**), 255 s (**d**), 330 s (**e**), 405 s (**f**), 480 s (**g**), and 800 s (**h**).

Figure 5 illustrates the solidification sequence on the outer side of the casting. During the initial stages of solidification, the outermost layers quickly solidify as cooling begins. These layers form a shell enveloping the melt. As shown in Figure 5b,d, the pattern of solidification zones originates from the thinnest regions and gradually spreads towards thicker areas. The top and bottom risers are typically the last areas to solidify, which precisely demonstrates that the design of risers in this study serves to compensate for shrinkage at critical locations of the casting.

**Figure 6:**
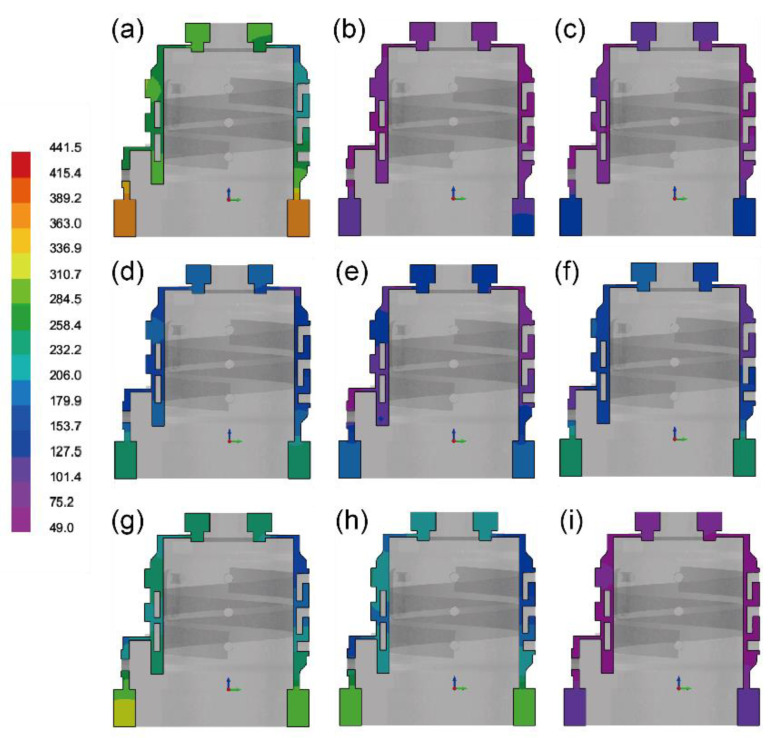
The time required for complete solidification at various locations of the casting in (**a**) Group 1, (**b**) Group 2, (**c**) Group 3, (**d**) Group 4, (**e**) Group 5, (**f**) Group 6, (**g**) Group 7, (**h**) Group 8, and (**i**) Group 9.

Figure 6 shows the time required for the temperature to reach the solidus at various locations of each group, elucidating the reasons behind the formation of this solidification sequence. It is observed from Figure 6a that at the upper and lower risers, due to their large volume and small heat dissipation area, these locations consistently exhibit the highest temperatures globally. This also implies that the portions solidifying last contribute to compensating for shrinkage in critical locations of the casting. The intermediate layers of the casting, on the other hand, solidify starting from the side away from the sprue. From the interface perspective, this is where the perimeter ratio is the largest, resulting in the fastest cooling rate, with solidification times significantly shorter than at other locations. According to the data in Figure 6, the time it takes for the bottom riser to reach the solidus line for Groups 1 to 9 are 369.3 s, 125.9 s, 138.2 s, 235.9 s, 182.6 s, 238.2 s, 313.1 s, 303.4 s, 110.5 s.

By comparing the simulated solidification time graphs of the nine different process parameter sets, it is observed that Groups 1, 4, 6, 7, and 8 exhibit longer solidification times. It can be inferred that the time for complete solidification of the melt is not strongly correlated with the pouring temperature. There is a significant range in the time for complete solidification at the same pouring temperature. However, when considering the heat transfer coefficient of the coating, a different trend emerges. Groups 1, 4, and 7 have a coating heat transfer coefficient of 50, resulting in significantly longer complete solidification times compared to Groups 3, 6, and 9, which have a coating heat transfer coefficient of 200. This indicates that the higher the heat transfer coefficient of the coating, the faster the dissipation of heat from the melt. As for the thermal conductivity of the sand, it also has a significant impact on the solidification process of the molten metal. In Groups 1, 6, and 8, silica dry sand is used, which possesses a lower thermal conductivity compared to the other groups. Particularly, when compared to Groups 2, 4, and 9, with the highest sand thermal conductivity, Groups 1, 6, and 8 exhibit a noticeable increase in complete solidification time. The phenomenon where Group 1, characterized by the lowest thermal conductivity of both sand and coating, exhibits the longest time for cooling to solidus, precisely illustrates the influence of both coating and sand on the solidification behavior of the melt.

**Figure 7:**
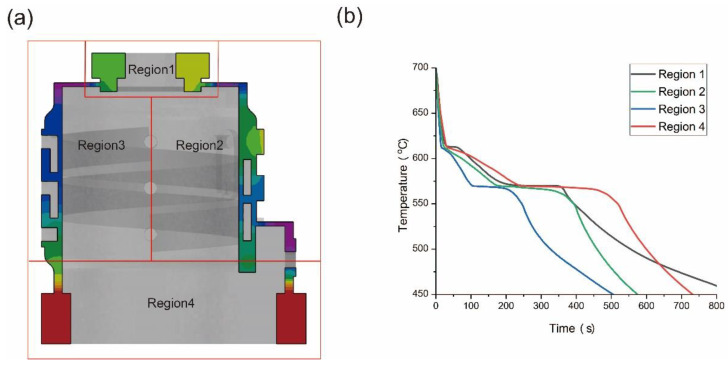
(**a**) Schematic diagram of regional division and (**b**) cooling curves of different regions.

As shown in Figure 7, based on the sequence of metal filling and solidification characteristics, we divided the casting into four regions. The area around the upper riser is designated as Region 1, while the sidewall closer to the inlet, the sidewall farther from the inlet, the lower riser, and their nearby areas constitute Regions 2, 3, and 4, respectively. Characteristic points were selected from the aforementioned regions, and the cooling curves were recorded, as shown in Figure 7. It can be observed that there is a significant difference in the solidification speed at different locations. The shortest time from the appearance of the solid phase to complete solidification occurs at the thin-walled area far from the inlet on the core of the casting, while the longest time is observed at the lower riser. Before complete solidification, the cooling rate of the melt is influenced by the relative surface area of the region. In areas with thin walls, the surface area for heat dissipation is large, and the volume storing heat is small, resulting in a faster cooling rate. Conversely, in thicker areas, the situation is the opposite. After complete solidification, the cooling rate in thicker regions becomes noticeably higher than in the initially solidified areas. This is due to a significant increase in the thermal conductivity of the aluminum alloy after solidification. Under the effect of temperature differences, heat from the thicker areas rapidly transfers to the thin-walled regions. Consequently, the cooling rate in the thin-walled areas after solidification is slower than in the thicker regions.

**Figure 8:**
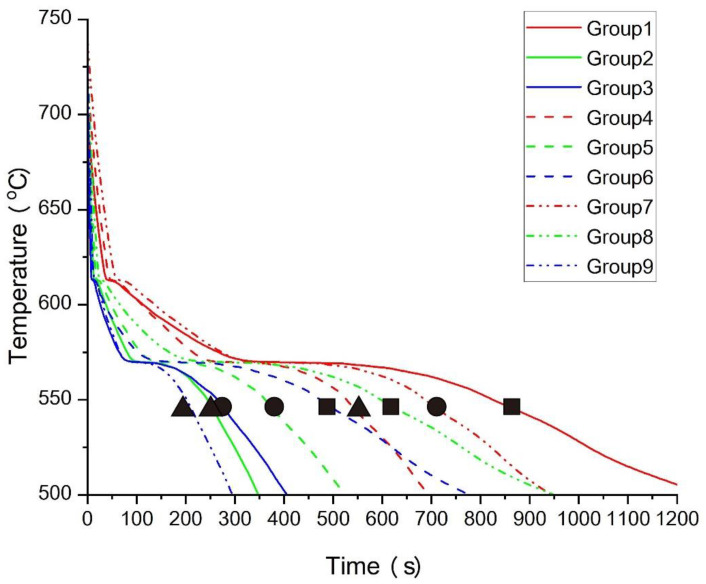
Cooling curves of Region 1 under nine different processes.

Figure 8 illustrates the cooling curves at the Region 1 position depicted in Figure 7, showcasing varying solidification rates across nine different processes. From Figure 8, it is evident that different processes have a significant impact on the cooling rate of the casting. The greater the solidification rate of the melt, the smaller the grain size of the casting, leading to corresponding improvements in its mechanical properties. In terms of pouring temperature, Groups 1, 2, and 3 are represented by solid lines at 700 °C; Groups 4, 5, and 6 are represented by dashed lines at 720 °C; and Groups 7, 8, and 9 are represented by double-dashed lines at 740 °C. When the pouring temperature is 720 °C, the cooling rate of the melt falls within a relatively small range. However, when the pouring temperature is 700 °C or 740 °C, the range of cooling rates is much larger. This indicates that although pouring temperature is one of the influencing factors on the solidification rate of the casting, its influence is not decisive. However, the pouring temperature can affect the fluidity of the metal, especially in lost-foam casting. Higher temperatures can facilitate more rapid and thorough decomposition of EPS, thereby achieving better filling results and improving the surface quality of the casting. However, excessively high pouring temperatures can increase the difficulty of metal treatment and the cost of smelting. Therefore, the optimal pouring temperature should be determined based on actual results and considerations of practicality. In terms of the heat transfer coefficient of the coatings, Groups 1, 4, and 7 are represented by the red color at 50; Groups 2, 5, and 8 are represented by the green color at 125; and Groups 3, 6, and 9 are represented by the blue color at 200. From Figure 8, it is evident that the heat transfer coefficient of the coatings has a decisive impact on the solidification behavior of the molten metal in aluminum alloy investment casting. With an increase in the heat transfer coefficient, there is a noticeable acceleration in the cooling rate. The selected position in Figure 8 represents the thinnest outer wall of the entire casting, making it the first location to solidify. The characteristics of aluminum alloys result in significantly higher thermal conductivity in the solid state compared to the liquid state. Therefore, fully solidified aluminum alloys play a role in accelerating the solidification rate of the surrounding liquid melt. Rapid solidification typically results in smaller grain sizes and higher mechanical properties of castings while also posing risks of shrinkage for castings with complex shapes. Therefore, the optimal heat transfer capability of coatings should be determined based on actual effectiveness. In terms of the thermal conductivity of sand, Groups 1, 6, and 8 are represented by squares, indicating a value of 0.5; Groups 3, 5, and 7 are represented by circles, indicating a value of 1; and Groups 2, 4, and 9 are represented by triangles, indicating a value of 2. From the figure, it can be observed that the thermal conductivity of the sand also significantly influences the cooling rate of the molten metal, although not as much as the heat transfer coefficient of the coatings. It can be seen that when solidification is occurring, that is, when the temperature of the melt is between the liquidus and solidus lines, the slope of the solidification curve is most affected by the thermal conductivity of the sand. Processes with lower thermal conductivity have longer solidification times compared to processes with higher thermal conductivity during this stage. The cooling process of the casting after complete solidification is also influenced by the thermal conductivity. In fact, Groups 4 and 6 even exhibit a phenomenon of intersecting cooling curves. The impact of the thermal conductivity of sand is evident on the alloy that is either in the process of solidification or has already solidified. Therefore, adjusting the thermal conductivity of the sand can fine-tune the solidification behavior of the casting to achieve the most reasonable cooling rate.

### 3.3. Prediction of Shrinkage Porosity Defects

**Figure 9:**
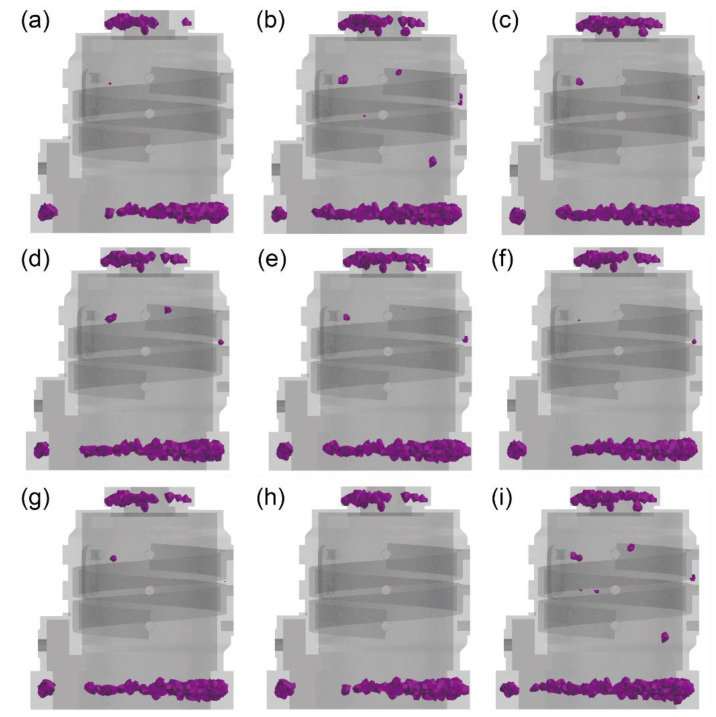
Illustrations of the statistical analysis of defects under (**a**) Group 1, (**b**) Group 2, (**c**) Group 3, (**d**) Group 4, (**e**) Group 5, (**f**) Group 6, (**g**) Group 7, (**h**) Group 8, and (**i**) Group 9.

Porosity is a common defect in aluminum alloy castings. During solidification, the volume of the aluminum alloy melt contracts due to changes in density. The volume contraction typically requires additional melt to replenish it. However, due to the direction of heat dissipation, the solidification sequence of the melt always proceeds from the outer regions towards the inner ones. This results in the last solidified areas lacking melt to compensate for volume shrinkage, leading to the formation of shrinkage porosity. Given the relatively thin wall thickness of the motor housing, most regions experience minimal volume contraction and are less prone to shrinkage porosity. However, in areas of small relative surface area, as depicted in the diagram, the cooling rate is often the slowest, making it susceptible to the formation of isolated liquid regions and the subsequent development of shrinkage porosity. Figure 9 illustrates the simulation results indicating the most susceptible locations for porosity (purple location) in the motor housing. It can be observed that porosity tends to concentrate primarily at the upper and lower risers. These areas represent the final solidification zones where no melt is available to compensate for shrinkage. As these locations are close to the casting, efforts should be made to minimize the volume of shrinkage to prevent the formation of porosity defects in critical areas of the casting. From Figure 9, it can be observed that only Groups 1, 6, and 8 exhibit defects at critical locations of the casting, with quantities not exceeding two and individual volumes not exceeding 0.02 cm^3^. Therefore, they represent feasible processes. Other processes, due to excessively rapid cooling rates or unreasonable solidification sequences, tend to exhibit shrinkage defects to varying degrees. This can lead to inadequate airtightness of the motor casing or inferior mechanical properties, making it difficult to meet product requirements or resulting in high scrap rates.

**Figure 10:**
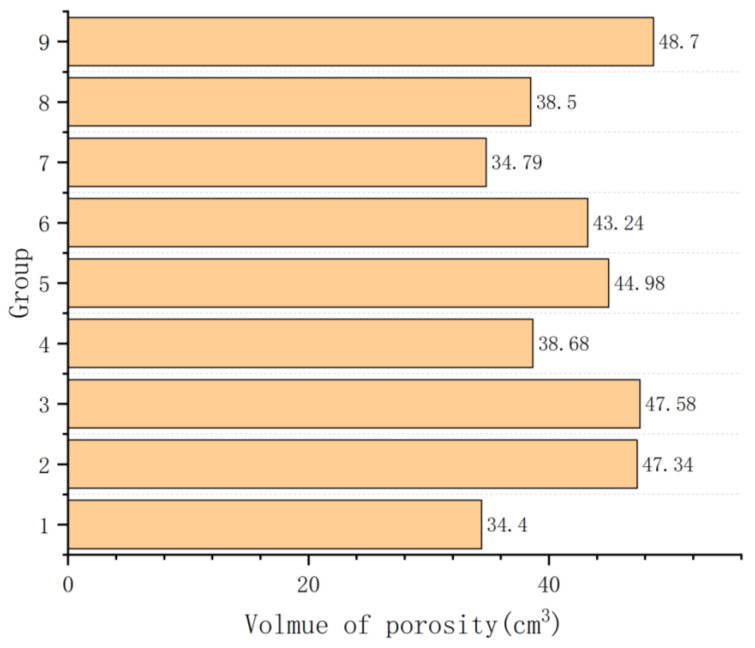
The statistical chart of defect volumes for each group.

Figure 10 illustrates the total volume of defects under different processes. Based on the extreme values in Figure 10, it can be observed that the interfacial heat transfer coefficient has the greatest impact on porosity, followed by the thermal conductivity of the sand and, finally, the pouring temperature. The defect volumes in Groups 2, 3, 5, 6, and 9 all exceed 40 cm^3^. These groups share a common characteristic of having higher interface heat transfer coefficients in their processes. It can be observed that during the solidification process of complex-shaped castings, excessively high interface heat transfer coefficients may lead to insufficient feeding and result in shrinkage defects. When the defect volume is too large, even if concentrated within the risers, it is possible for them to extend into the interior of the casting, thereby reducing the yield of finished castings. The influence of the sand’s thermal conductivity on casting defects is evident in Groups 1, 4, 7, and 8, where the total defect volume is less than 40 cm^3^. Groups 4 and 7, characterized by higher sand thermal conductivity, also exhibit larger shrinkage defects at critical locations of their castings. This indicates that although the excessively high thermal conductivity of the sand during the solidification process of the motor housing does not significantly increase the overall volume shrinkage, it does have an impact on the distribution of defects. The total volume of defects is minimal in Groups 1 and 8. Furthermore, based on the information from Figure 9, it is evident that defects in these two groups are concentrated at the positions of the upper and lower risers, ensuring high-quality castings. Therefore, it can be concluded that these two sets of process parameters are the most suitable. Considering the difficulty of alloy smelting and the cost of coatings, the optimal process parameters are determined to be Group 1 at a pouring temperature of 700 °C, dry sand thermal conductivity of 0.5, and interfacial heat transfer coefficient of 50.

## 4. Casting Verification

**Figure 11:**
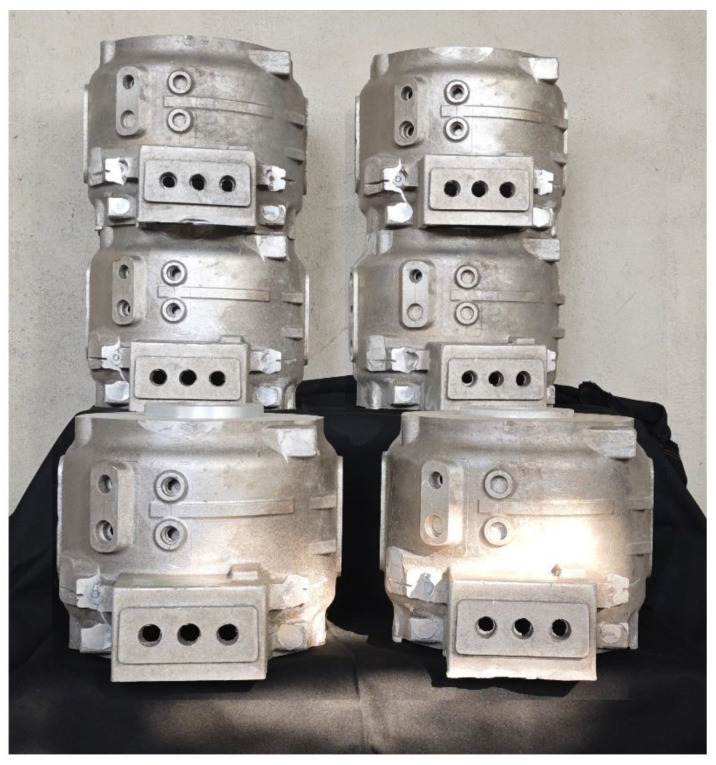
Castings produced with different process parameters.

**Figure 12:**
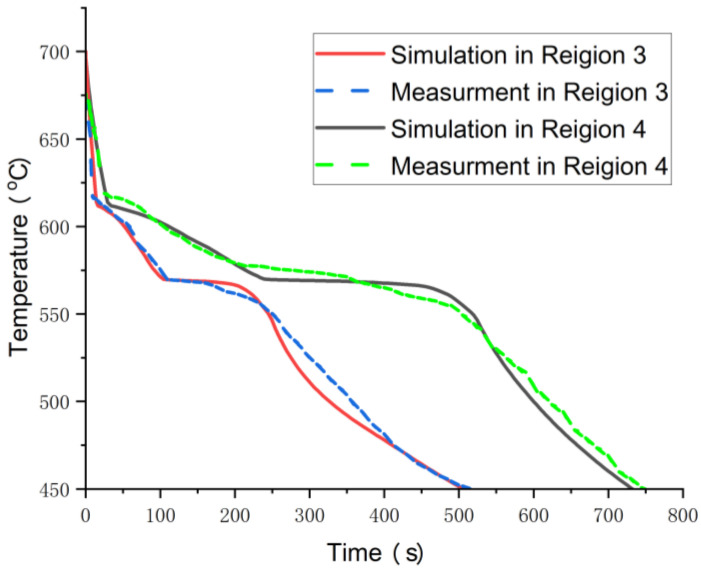
Comparison between the simulated cooling curve and the actual cooling curve.

**Figure 13:**
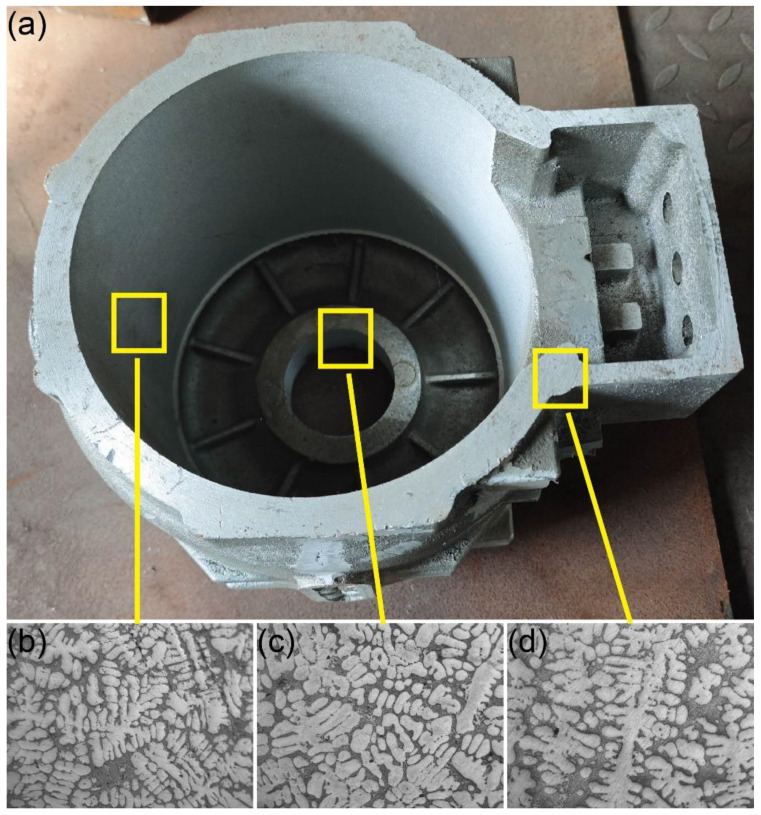
(**a**) Sampling locations of the casting and the microstructure of (**b**) wall, (**c**) upper riser, and (**d**) lower riser.

To validate the simulation results, casting experiments were conducted for each set of process parameters. Figure 11 displays the motor housing casting, where no defects or other quality issues were observed on the casting surface. Figure 12 illustrates the comparison between the simulated cooling curves and the experimentally measured cooling curves for Regions 3 and 4. The cooling rates between the two can be observed to be similar in Figure 12, with the curves during the initial cooling stage and eutectic reaction phase closely aligning. This suggests that the simulated results are representative of the actual solidification process. Figure 13a marks the sampling locations for critical parts of the casting. Figure 13b illustrates the microstructure of the thinnest part of the casting. Figure 13b,c show the microstructure of the casting at the location where it interfaces the upper and lower risers, respectively. Due to the adoption of the most optimal casting process, the casting remains intact overall, without any defects such as shrinkage. The microstructure of the casting is uniform, with no occurrence of inclusions or entrapments. The eutectic silicon is fine, with no occurrence of coarse silicon rods. Simultaneously, the microstructure of the casting varies according to the different cooling rates at different locations, as shown in Figure 7. In the faster-cooling Region 3, which corresponds to the sidewall of the motor casing, the secondary dendrite arm spacing is significantly smaller compared to it in Region 1 and Region 4. It is clear that the quality of the casting at these locations matches the simulation results, validating the accuracy and reliability of the simulation of the solidification process. Meanwhile, we sampled the same positions of castings from each group and measured their porosity using the density method. The porosity was determined by the difference between the sample density and the standard density. The results are shown in Table 3. This reaffirms that Group 1 represents the optimal process parameters.

## 5. Conclusions

This study determines the initial lost-foam casting process scheme for motor housing structures through structural analysis. Subsequently, the influence of solidification parameters on casting defects is analyzed through simulation of the flow field and temperature field. The following conclusions are drawn through analysis of defect volume statistics and distribution:

1. The filling process maintains a stable melt front interface, gradually diffusing filling with the inlet as the center. The filling speed is steady and influenced by the shape of the liquid metal front cross-section. When the pouring temperature ranges between 700 °C and 740 °C, the interfacial heat transfer coefficient of the coating ranges between 50 and 200, and the thermal conductivity of the sand ranges between 0.5 and 2, the filling is complete without any occurrences of underfill, entrapped gases, or inclusions.
2. Through analysis of the cooling curves under different processes, it has been determined that in lost-foam casting, the cooling conditions with the greatest impact on cooling speed are the heat transfer coefficient at the interface, followed by the thermal conductivity of the sand. The total solidification time of the melt significantly decreases with the increase in the interfacial heat transfer coefficient. The higher the thermal conductivity of the sand, the faster the cooling rate observed of the melt in the transitional temperature range. The effect of pouring temperature is minimal, only altering the starting position of the cooling curve.
3. There is a noticeable tendency for shrinkage at the risers, both upper and lower, ensuring adequate feeding to critical areas of the casting and avoiding defect formation. A high heat transfer coefficient at the interface significantly increases the overall volume of shrinkage, while the high thermal conductivity of the sand increases the proportion of defects at critical locations in the casting, both leading to a decrease in the yield rate of the casting. The optimal process parameters are determined to be a pouring temperature of 700 °C, dry sand thermal conductivity of 0.5, and interfacial heat transfer coefficient of 50.
4. Under the process parameters of Group 1 (pouring temperature of 700 °C, dry sand thermal conductivity of 0.5, and interfacial heat transfer coefficient of 50), the motor housing prepared by lost-foam casting achieves complete filling and excellent quality. The simulation results match the metallographic structure at the corresponding positions, confirming the accuracy of the simulation.

The results of this study provide a theoretical foundation for the preparation of complex-shaped and high-quality castings using lost-foam casting.

## Figures and Tables

**Table 1:** A356 parameters.

	Liquid	Solid
Density (kg/m^3^)	2520	2640
Conductivity (W/m·K)	80	170
Specific heat (kJ/kg·K)	1.04	0.896
Latent heat (kJ/kg)	431	/
Liquidus (°C)	613	/
Solidus (°C)	548	/

**Table 2:** Orthogonal experimental table.

Group	Pouring Temperature (°C)	Mold Conductivity (W/m·K)	Interface Heat Transfer Coefficient (W/m·K)
1	700	0.5	50
2	700	2	125
3	700	1	200
4	720	2	50
5	720	1	125
6	720	0.5	200
7	740	1	50
8	740	0.5	125
9	740	2	200

**Table 3:** The porosity rates of each group.

Group	1	2	3	4	5	6	7	8	9
Porosity(%)	0	1.6	1	1.3	1.3	0.2	0.5	0	1.8

## Data Availability

Data are contained within the article.
